# Dietary carbohydrates interact with *AMY1* polymorphisms to influence the incidence of type 2 diabetes in Korean adults

**DOI:** 10.1038/s41598-021-96257-z

**Published:** 2021-08-18

**Authors:** Dayeon Shin, Kyung Won Lee

**Affiliations:** 1 Department of Food and Nutrition, Inha University, Incheon, 22212 Republic of Korea; 2 Department of Home Economics Education, Korea National University of Education, Cheongju, 28173 Republic of Korea

**Keywords:** Type 2 diabetes, Epidemiology, Genetics, Diseases

## Abstract

The relationship between *AMY1* single nucleotide polymorphisms (SNPs), dietary carbohydrates, and the risk of type 2 diabetes is unclear. We aimed to evaluate this association using an ongoing large-scale prospective study, namely the Korean Genome and Epidemiology Study. We selected six genetic variants of *the AMY1* gene: rs10881197, rs4244372, rs6696797, rs1566154, rs1930212, and rs1999478. Baseline dietary data were obtained using a semi-quantitative food frequency questionnaire. Type 2 diabetes was defined according to the criteria of the World Health Organization and American Diabetes Association. During an average follow-up period of 12 years (651,780 person-years), 1082 out of 4552 (23.8%) patients had type 2 diabetes. Three *AMY1* SNPs were significantly associated with diabetes incidence among patients with carbohydrate intake > 65% of total energy: rs6696797, rs4244372, and rs10881197. In multivariable Cox models, Korean women with the rs6696797 AG or AA genotype had 28% higher incidence of type 2 diabetes (hazard ratio 1.28, 95% confidence interval 1.06–1.55) than Korean women with the rs6696797 GG genotype. We did not observe significant associations between *AMY1* SNPs, dietary carbohydrates, and diabetes incidence in Korean men. We conclude that *AMY1* genetic variants and dietary carbohydrate intake influence the incidence of type 2 diabetes in Korean women only. Korean women who are minor carriers of the *AMY1* rs6696797, rs4244372, and rs10881197 genotypes may benefit from a low-carbohydrate diet to prevent the future risk of type 2 diabetes.

## Introduction

Dietary carbohydrates may influence the development of type 2 diabetes by affecting blood glucose and insulin concentrations^1^. In a large prospective study of middle-aged Chinese women, rice was associated with an increased risk of type 2 diabetes^2^. In a 10-year prospective study of Japanese men, obese participants had a greater risk of type 2 diabetes when their carbohydrate intake was > 65% of the total energy intake (the acceptable macronutrient distribution range (AMDR) for carbohydrates for Japanese adults is 50–65%)^3^. In a meta-analysis of prospective cohort studies, the relative risk of type 2 diabetes was 1.11 (1.08–1.14) for each serving of white rice per day^4^. Multiple epidemiological studies indicate that dietary carbohydrate intake is positively associated with the risk of type 2 diabetes.

Salivary amylase is an enzyme that breaks down 1,4-alpha-glucoside bonds in oligosaccharides and polysaccharides, initiating dietary starch and glycogen digestion. Salivary amylase is encoded by the *AMY1* gene, which is involved in regulating dietary starch digestion and carbohydrate metabolism. Previous research indicated that *AMY1* copy number was positively associated with higher salivary amylase concentrations^5–8^. Additionally, individuals with higher salivary amylase concentrations showed lower postprandial blood glucose and high insulin levels after starch consumption^9^. Studies have found that individuals with more salivary amylase exhibit rapid starch breakdown, leading to faster and higher blood glucose responses following starch digestion^9^.

Populations with high-starch diets have more *AMY1* copies than populations with low-starch diets^5^. Starch consumption is dominant in agricultural societies, and this nutritional pressure has different effects on amylase activity^9^. During human evolution, the need to digest starchy food may have exerted selective pressure to increase *AMY1* copy number in populations consuming high-starch diets. Korea is one of the countries where rice is a major staple^10^, and the calorie consumption of 57% of Korean adults exceeds the upper recommended percentage of calories from carbohydrates (65%) from 2007 to 2012^11^. In recent decades, prevalence of type 2 diabetes has increased^12^. Low variation in *AMY1* copy number is correlated with high insulin resistance in healthy Korean men^13^. *AMY1* copy number and carbohydrate intake differentially influence the development of type 2 diabetes.

Single nucleotide polymorphisms (SNPs) near amylase genes are highly correlated with *AMY1* copy number^14^. Thus, nonsynonymous SNPs in the *AMY1* gene may be an additional genetic influence on amylase expression^5^. It is important to explore both SNPs and copy number variations when analyzing the genetic basis of disease risk^15^. Although the link between *AMY1* copy number variations and the risk of type 2 diabetes has been widely explored, few studies have examined the associations between *AMY1* genetic variants, dietary carbohydrate intake, and the incidence of type 2 diabetes. Understanding how these factors interact will provide insight regarding the role of genetic polymorphisms in type 2 diabetes etiology, with implications for further research on the physiological effect of *AMY1* SNPs. In particular, examining the role of *AMY1* genetic polymorphisms in the digestion of dietary carbohydrates is of great importance for improving our understanding of the type 2 diabetes pathophysiology. Using a prospective cohort study, we tested the hypothesis that *AMY1* genetic polymorphisms and carbohydrate intake interact to alter the incidence of type 2 diabetes.

## Results

### *AMY1* SNPs

Descriptions of six *AMY1* SNPs are presented in Table 1. *AMY1* rs6696797 was marginally associated with an increased risk for type 2 diabetes [adjusted odds ratio, 1.09; 95% confidence interval (CI) 0.99–1.20; *P* value = 0.089]. *AMY1* rs1999478 was significantly associated with glycated hemoglobin (β = 0.023, standard error = 0.011, *P* value = 0.035). *AMY1* SNPs and glucose levels were not significantly related.

**Table 1:** SNPs included in the study.

SNP	Chr	Minor allele	MAF	Type 2 diabetes		HbA1C (%)		Glucose (mg/dL)	
				AOR^1^	*P* value	β ± SE	*P* value	β ± SE	*P* value
rs6696797	1	A	0.370	1.09 (0.99–1.20)	0.089	0.014 ± 0.008	0.095	0.208 ± 0.188	0.269
rs4244372	1	A	0.362	1.08 (0.98–1.19)	0.144	0.017 ± 0.008	0.052	0.24 ± 0.188	0.202
rs10881197	1	G	0.373	1.08 (0.98–1.19)	0.133	0.014 ± 0.008	0.097	0.217 ± 0.188	0.248
rs1999478	1	A	0.165	1.06 (0.93–1.21)	0.367	0.023 ± 0.011	0.035	0.289 ± 0.245	0.239
rs1930212	1	G	0.200	1.05 (0.94–1.19)	0.392	0.004 ± 0.01	0.677	0.019 ± 0.224	0.934
rs1566154	1	G	0.279	1.03 (0.92–1.15)	0.602	− 0.016 ± 0.009	0.082	− 0.151 ± 0.203	0.457

Adjusted for age and sex. *P* value based on the additive genetic model.

*SNPs* single nucleotide polymorphisms, *Chr* chromosome, *MAF* minor allele frequency, *SE* standard error, *AOR* adjusted odds ratio.

### Participant baseline characteristics

During a follow-up period of 12 years on average, 1082 out of 4552 (23.8%) patients developed type 2 diabetes. General characteristics of the subjects grouped by carbohydrate intake are shown in Table 2. Participants with carbohydrate intake > 65% of total energy intake were more likely to live in Ansan, were non-smokers, older, more physically engaged, and less likely to drink than participants with carbohydrate intake ≤ 65%. Those with > 65% carbohydrate of total energy also had lower intake of total energy, total protein, animal protein, fat, sugar, and dietary fiber, and expectedly, had a higher intake of carbohydrates. We also found lower fasting glucose levels in participants with carbohydrate intake > 65% of total energy intake.

**Table 2:** General subject characteristics, stratified by carbohydrate intake.

	Carbohydrate intake ≤ 65% energy (n = 550)	Carbohydrate intake > 65% energy (n = 4002)	*P* value
Age (y)	48.8 ± 7.6	51.4 ± 8.5	< 0.0001
BMI (kg/m^2^)	24.5 ± 2.9	24.5 ± 3	0.971
**Residence**			
Ansung	190 (34.6)	1930 (48.2)	< 0.0001
Ansan	360 (65.5)	2072 (51.8)	
**Smoking behaviors**			
None	267 (48.6)	2465 (61.6)	< 0.0001
Past	106 (19.3)	629 (15.7)	
Current	177 (32.2)	908 (22.7)	
**Family history of diabetes**			
Yes	58 (10.5)	424 (10.6)	0.972
No	492 (89.5)	3578 (89.4)	
**Sex**			< 0.0001
Men	337 (61.3)	1844 (46.1)	
Women	213 (38.7)	2158 (53.9)	
Physical activity (MET-h/week)	154.5 ± 97.1	167.8 ± 103.3	0.004
Alcohol consumption (g/day)	15.2 ± 25.1	8.4 ± 19.9	< 0.0001
Total energy intake (kcal)	2297.9 ± 732	1960 ± 577.4	< 0.0001
Protein (g/day)	93 ± 33.9	60.4 ± 21.2	< 0.0001
Plant protein (g/day)	40.7 ± 14.7	39.5 ± 12.7	0.058
Animal protein (g/day)	52.3 ± 25.6	20.9 ± 12.7	< 0.0001
Fat (g/day)	56.4 ± 22	26.6 ± 13	< 0.0001
Carbohydrate (g/day)	348 ± 111.2	362.3 ± 106.6	0.004
Sugar (g/day)	48.3 ± 25.3	40 ± 28.2	< 0.0001
Dietary fiber (g/day)	16.8 ± 7.8	14.8 ± 8.7	< 0.0001
HbA1c (%)	5.6 ± 0.4	5.6 ± 0.4	0.671
Fasting glucose (mg/dL) (n = 3987)	84.1 ± 8.9	83.1 ± 8.7	0.009
Insulin (µIU/mL) (n = 3987)	7.2 ± 3.6	7.5 ± 4.5	0.12
HOMA-IR (n = 3987)	1.5 ± 0.8	1.5 ± 1	0.382

Data are presented as mean ± standard deviation or n (%).

*P* values are from chi-square tests for categorical variables and t-tests for continuous variables.

*SD* standard deviation, *MET* metabolic equivalent task, *HOMA-IR* homeostatic model assessment of insulin resistance.

### Relationship between ***AMY1*** SNPs, dietary carbohydrates, and incidence of type 2 diabetes in men

Table 3 shows the adjusted HRs and corresponding 95% CIs for the prospective association between six *AMY1* SNPs and diabetes incidence by carbohydrate intake in Korean men. After adjusting for age, residence area, education level, smoking status, alcohol consumption, physical activity, BMI, and family history of diabetes, we did not observe significant associations between individual *AMY1* SNPs and the incidence of type 2 diabetes in either low- or high-carbohydrate groups in men.

**Table 3:** Adjusted hazard ratios (and 95% CIs) for associations between *AMY1* SNPs and incidence of type 2 diabetes, stratified by carbohydrate intake in Korean men.

Men with % energy carbohydrate intake ≤ 65%			Men with % energy carbohydrate intake > 65%		
	HR	(95% CI)		HR	(95% CI)
**rs6696797**			**rs6696797**		
GG	1.00		GG	1.00	
AG + AA	1.14	(0.74–1.76)	AG + AA	1.08	(0.90–1.30)
**rs4244372**			**rs4244372**		
TT	1.00		TT	1.00	
AT + AA	1.11	(0.72–1.70)	AT + AA	1.08	(0.90–1.30)
**rs10881197**			**rs10881197**		
CC	1.00		CC	1.00	
GC + GG	1.15	(0.74–1.77)	GC + GG	1.07	(0.89–1.29)
**rs1999478**			**rs1999478**		
CC	1.00		CC	1.00	
AC + AA	1.23	(0.80–1.89)	AC + AA	1.02	(0.84–1.24)
**rs1930212**			**rs1930212**		
AA	1.00		AA	1.00	
GA + GG	0.92	(0.60–1.43)	GA + GG	1.02	(0.85–1.23)
**rs1566154**			**rs1566154**		
AA	1.00		AA	1.00	
GA + GG	0.76	(0.50–1.16)	GA + GG	1.06	(0.88–1.26)

Adjusted for age (years), area of residence (Ansan or Ansung), education level [elementary school or lower (< 7 years completed), middle/high school (7–12 years), college or higher (> 12 years)], smoking status (never, former smoker, current smoker), alcohol consumption (g/day), physical activity [metabolic equivalent task (MET)-h/week], body mass index (BMI; kg/m^2^), and family history of diabetes (self-reports: yes, no).

*HR* hazard ratio, *CI* confidence intervals.

### Relationship between ***AMY1*** SNPs, dietary carbohydrates, and incidence of type 2 diabetes in women

Table 4 presents the adjusted HRs and corresponding 95% CIs for associations between *AMY1* SNPs and incidence of type 2 diabetes by carbohydrate intake among Korean women. Three *AMY1* SNPs were significantly associated with the incidence of type 2 diabetes in women with > 65% carbohydrate intake from energy: rs6696797, rs4244372, and rs10881197. In the > 65% group, women with the rs6696797 AG or AA genotype had a 28% higher incidence of type 2 diabetes (HR 1.28, 95% CI 1.06–1.55) than women with the rs6696797 GG genotype. Women with the rs4244372 AT or AA genotype had a 29% higher incidence of type 2 diabetes than those with the TT genotype (HR 1.29, 95% CI 1.07–1.56). Women with the rs10881197 GC or GG genotype had a 30% greater incidence of type 2 diabetes than those with the CC genotype (HR 1.30, 95% CI 1.08–1.57).

**Table 4:** Adjusted hazard ratios (and 95% CIs) for associations between *AMY1* SNPs and incidence of type 2 diabetes, stratified by carbohydrate intake in Korean women.

Women with % energy carbohydrate intake ≤ 65%			Women with % energy carbohydrate intake > 65%		
	HR	(95% CI)		HR	(95% CI)
**rs6696797**			**rs6696797**		
GG	1.00		GG	1.00	
AG + AA	0.76	(0.39–1.46)	AG + AA	1.28	(1.06–1.55)
**rs4244372**			**rs4244372**		
TT	1.00		TT	1.00	
AT + AA	0.80	(0.41–1.55)	AT + AA	1.29	(1.07–1.56)
**rs10881197**			**rs10881197**		
CC	1.00		CC	1.00	
GC + GG	0.76	(0.39–1.46)	GC + GG	1.30	(1.08–1.57)
**rs1999478**			**rs1999478**		
CC	1.00		CC	1.00	
AC + AA	0.69	(0.32–1.48)	AC + AA	1.14	(0.94–1.39)
**rs1930212**			**rs1930212**		
AA	1.00		AA	1.00	
GA + GG	0.95	(0.49–1.85)	GA + GG	1.06	(0.88–1.27)
**rs1566154**			**rs1566154**		
AA	1.00		AA	1.00	
GA + GG	0.81	(0.42–1.54)	GA + GG	1.00	(0.83–1.19)

Adjusted for age (years), area of residence (Ansan or Ansung), education level [elementary school or lower (< 7 years completed), middle/high school (7–12 years), college or higher (> 12 years)], smoking status (never, former smoker, current smoker), alcohol consumption (g/day), physical activity [metabolic equivalent task (MET)-h/week], body mass index (BMI; kg/m^2^), and family history of diabetes (self-reports: yes, no).

*HR* hazard ratio, *CI* confidence intervals.

### Cumulative incidence of type 2 diabetes by ***AMY1*** rs10881197 genotypes and dietary carbohydrates

**Figure 1:**
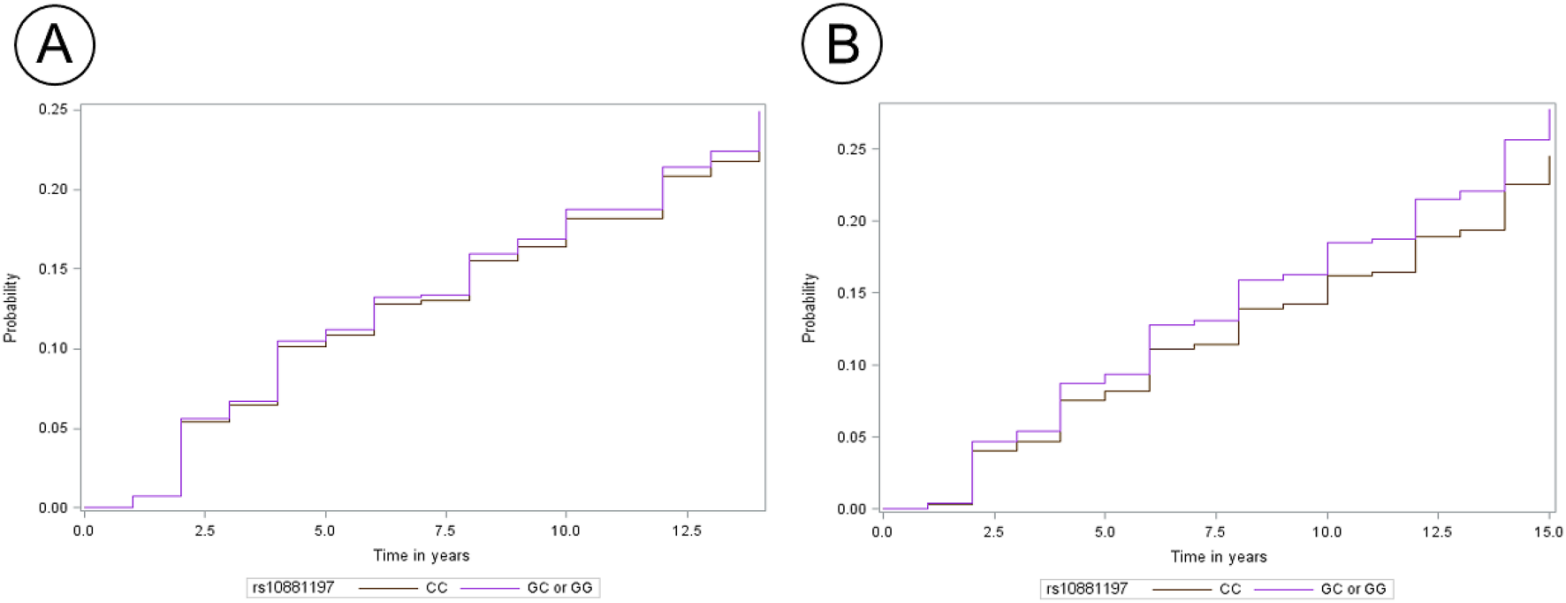
Cumulative incidence of type 2 diabetes by carbohydrate intake. (**A**) Comparison of cumulative incidence between rs10881197 CC and rs10881197 GC or GG genotypes for carbohydrate intake ≤ 65% of total energy intake. (**B**) Comparison of cumulative incidence between rs10881197 CC and rs10881197 GC or GG genotypes for those with carbohydrate intake > 65% of total energy intake.

Figure 1 shows the Kaplan–Meier plot of the cumulative type 2 diabetes incidence, grouped by *AMY1* rs10881197 genotypes (CC vs. GC or GG) and carbohydrate intake groups (≤ 65% or > 65% energy). Among the carbohydrate intake ≤ 65% group, cumulative incidence of type 2 diabetes was 10%, 17.9%, and 23.9% in 5, 10, and 15 years, respectively, for individuals with the rs10881197 CC genotype. For those with the rs10881197 GC or GG genotype, cumulative incidence of type 2 diabetes was 12%, 19.2%, and 24.1% in 5, 10, and 15 years, respectively. Among the carbohydrate intake > 65% group, the cumulative incidence of type 2 diabetes was 8.1%, 16.3%, and 21.9% in 5, 10, and 15 years for those with the rs10881197 CC genotype, respectively. For the rs10881197 GC or GG genotype, the cumulative incidence of type 2 diabetes was 9.7%, 18.7%, and 24.9% in 5, 10, and 15 years, respectively. Overall, the cumulative incidence of type 2 diabetes was the highest in participants who had a carbohydrate intake > 65% and carried the rs10881197 GC or GG genotype.

## Discussion

In this average of 12-year, prospective population-based cohort study of middle-aged Korean adults, we demonstrated that carriers of minor alleles in *AMY1* (rs6696797, rs4244372, and rs10881197) had significantly elevated incidence of type 2 diabetes if they were women who obtained > 65% of their total energy intake from carbohydrates. To the best of our knowledge, this is the first study to test the hypothesis that *AMY1* genetic variants, coupled with higher carbohydrate intake, are associated with a higher incidence of type 2 diabetes. Notably, we only observed these significant associations in women and not in men, suggesting a potential sex-specific mechanism related to sex hormones or sex chromosome complement^16^. The Toronto Nutrigenomics and Health Study reported that *AMY1* rs10881197 and *AMY1* copy numbers were both associated with lower energy intake among 20–29-year-old Caucasian adults^17^. Thus, *AMY1* SNPs may contribute to starch and energy intake.

Perry et al.^5^ reported that although *AMY1* protein expression is correlated with *AMY1* copy number, the latter does not fully explain a considerable degree of variation in the expression of the *AMY1* protein (R^2^ = 0.351). Therefore, other genetic factors, such as regulatory-region SNPs, could influence *AMY1* expression. Both *AMY1* copy number and *AMY1* SNPs may explain between-individual differences in *AMY1* protein expression, and accordingly, the reason for some individuals having a higher risk of developing type 2 diabetes than others. Here, we observed the greatest disease incidence in women who consumed high-carbohydrate diets and possessed the A allele of rs6696797, A allele of rs4244372, and G allele of rs10881197. In healthy, non-obese individuals, those with low amylase activity did not have increased insulin levels before glucose absorption began, whereas individuals with high amylase activity had lower postprandial blood glucose concentrations^9^. Evolutionarily, individuals with high *AMY1* copy numbers and salivary amylase activity have more rapid and efficient starch digestion mechanisms, which ensure the quick availability of blood glucose for extreme energy expenditure activities like farming or hunting^9^.

A previous study reported that high *AMY1* copy number is associated with enhanced glucose absorption following an oral glucose load^19^. The authors suggested that the co-evolution of high *AMY1* copy number with increased carbohydrate resulted in an enhanced salivary digestion of starch to maltose, followed by conversion to glucose. This enhancement optimized the efficiency of glucose absorption across the upper gastrointestinal tract. Thus, both genetic and dietary factors are implicated in the development of type 2 diabetes. Here, we found that the cumulative incidence of type 2 diabetes was highest at year 15 for participants regardless of the rs10881197 genotypes, but significant difference in cumulative incidence by rs10881197 genotypes (CC vs. GC or GG) was found only in the high carbohydrate-intake group. Cumulative incidence at year 15 was lowest among the participants with the rs10881197 CC genotype and their counterparts in the low-carbohydrate-intake group. This result emphasizes the combined effects of *AMY1* genotypes and dietary carbohydrates on the incidence of type 2 diabetes.

Type 2 diabetes is strongly linked with obesity^9^, a major cause of insulin resistance^20,21^. Hence, a growing body of literature has investigated the relationship between *AMY1* copy number^22–28^ and obesity, but the outcome has been inconsistent, either failing to find any associations^22–24^ or identifying an inverse relationship^25–28^. These inconsistencies may be partially due to between-study differences in techniques for evaluating the *AMY1* copy numbers and in the racial/ethnic compositions of the subject populations. Although the link between *AMY1* copy numbers and obesity remains uncertain, *AMY1* copy number is associated with salivary amylase concentrations^6^, and this relationship may contribute to individual differences in dietary starch intake. Chronically elevated blood glucose, induced by high starch intake, may cause hormonal, receptor, and physiological changes that would eventually result in type 2 diabetes^9^. Furthermore, a diet rich in sugars impaired cardiac systolic and diastolic function in the mouse^29^. Besides SNPs in *AMY1* gene, several SNPs in susceptibility genes have been associated to insulin sensitivity or insulin resistance. As genes involved in adipose tissue metabolism may influence insulin sensitivity, the polymorphisms of adiponectin gene (−11,391G>A, − 11,377C>G, and +45T>G) were associated with insulin resistance state in overweight/obese children^18^. It is significant to explore a wide variety of genetic factors for insulin resistance for future studies.

Our study has several limitations. First, we lacked the data to analyze salivary amylase concentration; thus, we could not further explore the linkage between *AMY1* SNPs and salivary amylase concentrations. Additionally, the study was limited to participants who were over 40 years old and living in Ansan or Ansung. Therefore, our findings may not be applicable to the general Korean population.

Nevertheless, this study has several strengths. To our knowledge, it is the first to examine the association between *AMY1* genetic variants and the incidence of type 2 diabetes after the stratification of the study population based on carbohydrate intake, using a 16-year prospective follow-up design. We were also able to control for multiple confounding variables because KoGES provided relevant health- and disease-related phenotypic data^30^. Finally, exploring the genetic basis of type 2 diabetes risk requires measuring all forms of genetic variation, including SNPs. Accordingly, this work contributes to expanding the existing knowledge on the combined role of *AMY1* and dietary carbohydrates on the development of type 2 diabetes.

In conclusion, we demonstrated that the *AMY1* genetic variants and dietary carbohydrate intake influenced the incidence of type 2 diabetes among Korean women. The underlying mechanisms linking the genes, diet, and disease remain unclear, but our findings suggest that *AMY1* SNPs and dietary factors are both important for type 2 diabetes etiology. This study provides a sound empirical basis for screening individuals who are minor carriers of *AMY1* (rs6696797, rs4244372, and rs10881197) and have a high-carbohydrate diet, to detect future risk of type 2 diabetes.

## Research design and methods

### Study design and participants

We used data from the Ansan-Ansung Cohort Study of the Korean Genome and Epidemiology Study (KoGES), an ongoing large-scale prospective study conducted by the Korea National Institute of Health^30^. The Ansan-Ansung study was initiated in 2001–2002 (baseline) to explore dietary and lifestyle factors that affect chronic diseases in the Korean population. It recruited 10,030 adults (40–69 years old) who resided in Ansan (urban) and Ansung (rural). Participants were followed up bi-annually, and we included their follow-up data collected until 2012.

**Figure 2:**
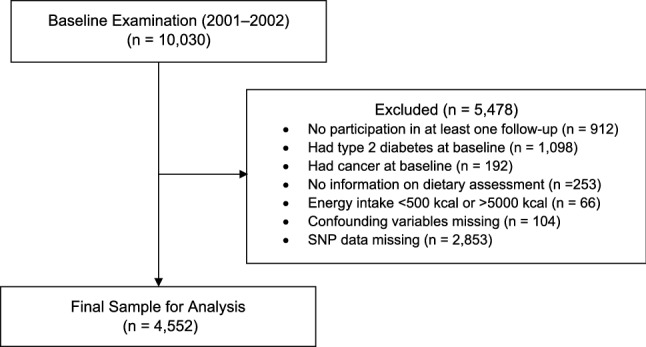
Flowchart of the study participants.

From 10,030 participants at baseline examination (2001–2002), we excluded those who did not participate during the follow-up at least once (n = 912), had a diagnosis of type 2 diabetes at baseline (n = 1098), had a diagnosis of cancer (n = 192), lacked dietary information (n = 253), had energy intake < 500 kcal/day or > 5000 kcal/day (n = 66), had missing data for confounding variables (n = 104), or had no SNP data (n = 2853). The final analytical sample comprised 4552 individuals (Fig. 2). The KoGES study was reviewed and approved by the Institutional Review Board of the Korea Centers for Disease Control and Prevention. All participants enrolled in the study voluntarily and all gave written-informed consent. All study methods and protocols were conducted in accordance with the relevant institutional guidelines and regulations. The protocol was reviewed and approved by the Institutional Review Board of Inha University on January 31, 2020 (IRB No. 2001291A).

### Dietary assessment

Dietary data were collected by well-trained interviewers at baseline, using a 103 item semi-quantitative food frequency questionnaire (FFQ). This validated FFQ was developed to assess the usual dietary intake of Korean adults who participated in the KoGES^31^. All participants were asked how often they consumed each food item during the previous year. They could give nine possible responses ranging from never or seldom to ≥ 3 times per day^31^. To calculate the typical intake of foods and nutrients, including carbohydrates, consumption frequency for each unit of food was multiplied by the nutrient content of each food item, by referring to a nutrient database (CAN-Pro 2.0) developed by the Korean Nutrition Society^32^. For this study, carbohydrate intake (g/day) and percentage contribution in terms of energy (% energy) were assessed using the FFQ.

### Assessment of type 2 diabetes

During the biennial follow-up examination, type 2 diabetes was categorized as fasting blood glucose ≥ 126 mg/dL after at least 8 h of fasting and 2 h plasma glucose level of 75 g oral glucose tolerance test ≥ 200 mg/dL, in accordance with the criteria of the World Health Organization^33^ and American Diabetes Association^34^. Other acceptable criteria were diagnosis of diabetes by a physician, use of insulin treatment, or use of diabetic medication.

### Genotyping and imputation

Imputed genotypes were produced by the Korea BioBank Array Project (or KoreanChip, KCHIP) for the Korean population. Genetic data were available through the KCHIP consortium^35^. The KCHIP, containing about 833,535 SNPs specific to the Korean population, was designed by the Center for Genome Science, Korea National Institute of Health, Korea (4845-301, 3000-3031). Standard quality control procedures (Hardy–Weinberg equilibrium P ≥ 1.0 × 10^−6^, call rate ≥ 95%, and INFO ≥ 0.8) were used. Genetic data were imputed using SHAPEIT v2-IMPUTE v2, with the 1000 Genomes Project phase 3 reference provided by the Center for Genome Science, Korea National Institute of Health^35^.

### Statistical analyses

Six genetic variants of *AMY1* were selected: rs10881197 (minor allele, G), rs4244372 (A), rs6696797 (A), rs1566154 (G), rs1930212 (G), and rs1999478 (A) (Table 1). Genetic analysis was performed in PLINK (version 1.90 beta, https://www.cog-genomics.org/plink/1.9).

Men and women were analyzed separately. Subjects were categorized into two groups: ≤ 65% carbohydrate-derived energy (maximum AMDR for carbohydrates) and > 65%, according to the Dietary Reference Intakes for Koreans 2020^36^. Baseline sociodemographic and lifestyle characteristics of participants were compared using chi-square tests for categorical variables and ANOVA for continuous variables. Hazard ratios (HRs) and 95% confidence intervals (CIs) for the incidence of type 2 diabetes were estimated with Cox proportional hazard models, using individual *AMY1* SNPs under a dominant genetic model stratified by carbohydrate intake (≤ 65% or > 65%). Covariates were age (years), area of residence (Ansan or Ansung), education level [elementary school or lower (< 7 years of school completed), middle/high school (7–12 years), college or higher (> 12 years)], smoking status (never, former smoker, current smoker), alcohol consumption (g/day), physical activity [metabolic equivalent task (MET)-h/week], body mass index (BMI; kg/m^2^), and family history of diabetes (determined from self-reports: yes, no). For each *AMY1* SNP, we further employed Kaplan–Meier plots to examine the cumulative incidence of type 2 diabetes during follow-up periods, stratified by carbohydrate intake (≤ 65% and > 65%). All statistical analyses were performed in SAS software (version 9.4; SAS Institute, Cary, NC, USA). Significance was set at *P* value < 0.05 (two-sided).
